# The Impact of Pelvic Floor Muscle Strengthening on the Functional State of Women Who Have Experienced OASIS After Childbirth

**DOI:** 10.3390/medicina61010022

**Published:** 2024-12-27

**Authors:** Atėnė Simanauskaitė, Justina Kačerauskienė, Dalia Regina Railaitė, Eglė Bartusevičienė

**Affiliations:** Lithuanian University of Health Sciences, 44307 Kaunas, Lithuania; justina.kacerauskiene@lsmu.lt (J.K.); daliaregina.railaite@lsmu.lt (D.R.R.); egle.bartuseviciene@lsmu.lt (E.B.)

**Keywords:** pelvic floor muscle, training, OASIS, postpartum, incontinence

## Abstract

*Background and Objectives:* The primary objective of this study was to assess the impact of pelvic floor muscle (PFM) strengthening on the pelvic floor function in women who have experienced OASIS two years after delivery, and the secondary objective was to educate women about PFM strengthening and instruct them on the correct way to exercise. *Methods and Materials:* A prospective case-control study was conducted. The participants were divided into two groups: the case group (women who experienced OASIS) and the control group (women who did not experience perineal tears but had similar obstetric-related data to the case). Women were invited for a gynecological exam, PFM assessment, and consultation on PFM training. Women in the case group had three consultations, and women in the control group had two. Women were presented with four sets of questions about pre-pregnancy condition and questions related to UI and FI after delivery. Results were considered significant when *p* < 0.05. *Results:* OASIS were detected in 13 (0.4%) women in 2021. Risk factors for OASIS were found to be fetal macrosomia (*p* = 0.012), fetal occiput posterior position (*p* = 0.001), and epidural analgesia (*p* = 0.003). After one year of performing PFM strengthening exercises, some women in the study group exhibited stronger PFM contractions (*p* = 0.076), while others held the contracted PFM for a longer time (*p* = 0.133). UI affected women in the control group more often (*p* = 0.019). Two years after delivery, gas incontinence was mentioned significantly more frequently in the case group (*p* = 0.019). One year after initial consultation, gas incontinence was also more significantly common in the case group (*p* = 0.037). *Conclusions:* This study found that PFM strengthening exercises significantly improved pelvic floor function in women who experienced OASIS two years after delivery. Participants exhibited stronger PFM contractions and an increased ability to maintain these contractions. Women reported a better understanding of PFM exercises and proper techniques.

## 1. Introduction

Over 85% of women experiencing vaginal childbirth will experience some level of perineal tearing [1]. Among all vaginal deliveries, 0.6–11% lead to obstetric anal sphincter injuries (OASIS), a third- or fourth-degree perineal tear [1,2]. The incidence of perineal tears tends to decrease with subsequent birth, dropping from 90.4% in first-time mothers to 68.8% in women who have had multiple vaginal deliveries [3]. Women who experience OASIS during childbirth are at risk of experiencing pelvic floor dysfunction in the future, characterized by urinary incontinence (UI), fecal incontinence (FI), pelvic organ prolapse (POP), altered sexual function, or chronic pelvic pain [4].

According to the recommendations of the International Continence Society [5], the European Association of Urology [6], and other organizations, pelvic floor muscle (PFM) training is the primary choice for both the prevention and treatment of pelvic floor dysfunction. The strengthening of the PFM can be initiated as early as possible after childbirth, as it reduces perineal pain and promotes faster tissue healing and functional recovery [5]. However, women may choose to commence PFM exercises later, at their convenience (for example, after 1–2 years after delivery or even later) [7]. Scientific studies show that information about PFM and targeted PFM strengthening are particularly crucial for women who are at risk for pelvic floor dysfunction [8].

Unfortunately, in Lithuania, women still do not have enough information about PFM training in the postpartum period. Consequently, if they do not actively seek information, they may not engage in PFM exercises [9]. Therefore, the primary objective of this study was to inform and educate women about PFM strengthening and its significance and to instruct them on the correct way to exercise. The secondary objective of this study was to assess the impact of PFM strengthening on the pelvic floor function in women after OASIS two years after delivery.

## 2. Materials and Methods

A prospective case-control study was conducted at the Lithuanian University of Health Sciences Hospital in Kauno klinikos (LUHS KK) Department of Obstetrics and Gynecology in 2023. Women who experienced a high-degree perineal tear in 2021 were involved in the study. Data were collected from birth records, case histories, and the hospital information system. The selected participants were assessed in two groups: the study group (women after OASIS) and the control group (women who did not experience 3rd- or 4th-degree perineal tears but gave birth in the same year at a very similar time and whose pregnancy- and delivery-related data were similar to those of women in the study group).

Approval for the study was obtained from the Kaunas Regional Biomedical Research Ethics Committee under the approval number BE–2–3.

In 2023, a total of 2902 women delivered at the Department of Obstetrics and Gynecology, a tertiary teaching center of the Hospital of Lithuanian University of Health Sciences, with 13 (0.4%) of those women who experienced OASIS being invited to participate in this study.

Sociodemographic and anthropometric data, obstetrical history, and pregnancy-related information were collected from medical documentation. All perineal tears were evaluated in delivery wards by examining the perineum after delivery: 1st-degree tear—superficial tear involving the vaginal mucosa, perineal skin, and superficial fascia; 2nd-degree tear—deep perineal tear not reaching the anal sphincter; 3rd-degree tear—partially or completely tearing the anal sphincter (subtypes: 3A—external sphincter torn up to 50% of muscle thickness, 3B—complete external sphincter tear, and 3C—torn internal sphincter); 4th-degree (IV°) perineal tear—involving the anal mucosa and potentially extending to the rectal mucosa [1].

Two years after delivery, women were invited by phone to enter the study. They were invited for a gynecological exam, assessment of PFM function, and consultation on PFM training. Women in the case group had three consultations: the initial consultation (two years after the delivery that was in 2021) and two later consultations 1 month and 1 year after the initial consultation. Women in the control group had two consultations: the initial consultation and another 1 year after it. Participants in the case group were contacted twice at two-week intervals between the initial and the second consultation by phone, with the purpose of reminding them about PFM training benefits and answering any arising questions.

During the initial consultation, a gynecological exam was performed, PFM strength was assessed, and training in PFM exercises was provided. During the consultation, the functional status of the PFM was evaluated using a modified Oxford scale. The PFM contraction was rated on a scale of 0 to 5 points, according to the modified Oxford scale: 0—no PFM contraction; 1—minor muscle ‘flicker’; 2—weak muscle contraction; 3—moderate muscle contraction; 4—good muscle contraction; and 5—strong voluntary contraction with resistance [10]. The PFM training program was nearly identical for all participants, with only a few individuals receiving personalized programs based on their PFM condition. Moreover, all questions about PFM strengthening were answered.

All women were taught how to perform PFM strengthening exercises and were suggested to exercise 2 times per day.

During the initial consultation, women were presented with four sets of questions regarding their pre-pregnancy condition, questions related to UI (using the International Consultation on Incontinence Questionnaire Short Form—ICIQ–SF), questions related to FI (using the Wexner Fecal Incontinence Scale), and questions related to quality of life (using the Pelvic Floor Impact Questionnaire—PFIQ–7). During the second consultation, a gynecological exam was performed, the PFM condition was evaluated, and questions about PFM training were answered. In the third consultation, a gynecological exam was performed, the PFM condition was assessed, questionnaires (ICIQ-SF, Wexner Fecal Incontinence scale, and PFIQ-7) were repeated, and experiences about how the exercise routine progressed over the year and challenges in remembering and performing the exercises were discussed.

Data were collected, processed, and analyzed using the statistical data analysis package IBM SPSS Statistics 23. The statistical association between qualitative data from two random samples was assessed using the chi-square (χ^2^) test, and for quantitative measurements, Student’s *t*-tests were applied for comparisons. Results were considered significant when *p* < 0.05. Percentages, frequencies, and means with standard deviations were calculated for the characteristics.

## 3. Results

**Figure 1:**
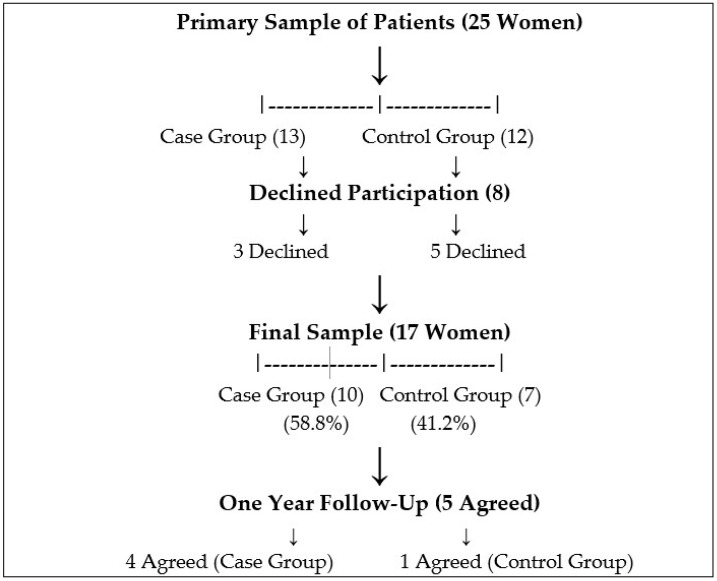
The diagram of the study sample formation.

During the study period from 1 January 2021 to 31 December 2021, 3098 women gave birth at the Department of Obstetrics and Gynecology of LUHS KK. A total of 13 (0.4%) of the women experienced OASIS. The primary sample of patients was 25 women (13 women in the case group and 12 in the control group). However, eight participants (three women from the case group and five from the control group) declined to participate in the study. Therefore, the final sample of participants consisted of 17 women: the case group consisted of 10 patients (58.8%), while the control group consisted of 7 women (41.2%). One year after the initial consultation, women were invited to the consultation once again. However, only five of them (four from the case group and one from the control group) agreed to come for a consultation (Figure 1).

The median age of the participants was 33 years. Women in the control group were statistically significantly younger (*p* = 0.024) than women in the case group. Sociodemographic data are presented in Table 1. Approximately one-third of women were classified as overweight both before and after pregnancy in the case group.

Pregnancy- and delivery-related information were compared. Fetal macrosomia was more often diagnosed in the case group (*p* = 0.012). Women included in the study most frequently gave birth at 39 weeks of pregnancy. Labor was induced more often in the case group (*p* = 0.036). The average duration of the first and second stages of labor for women in the case group was statistically significantly longer than in the control group (*p* = 0.038, and *p* = 0.043). Respectively, risk factors for OASIS are presented in Table 2.

The complaints related to pelvic floor dysfunction during the initial consultation (two years after delivery) were investigated. Almost half (47.1%) of the women enrolled in the case group reported at least one complaint associated with pelvic floor dysfunction. Stress UI was more common in the case group (*p* = 0.019). The incidence of other complaints, such as other types of UI, FI, or dyspareunia, did not differ in both groups.

After one month of training, 70% of women (*n* = 12) exercised regularly (10 women from the case group and 2 from the control group) or two times per day, while the other five women (all from the control group) exercised irregularly or one time per day. After one year of PFM training, only one-third of women in both groups performed PFM exercises regularly (one or three times per day), and the majority of women were in the case group.

The functional status of women’s pelvic floor was assessed using the modified Oxford scale. The contraction of the pelvic floor was rated on a scale from 0 to 5, with no statistically significant differences identified in both groups. Before performing PFM strengthening exercises, women in both groups contracted with a strength of 2 or 3 points. The median point of the contracted strength in the case group was 2 [min–max: 1–4], while in the control group, it was 3 [min–max: 1–5]. The impact of PFM training on women who have experienced OASIS during delivery was evaluated after one month and after one year of performing PFM strengthening exercises. Although no statistically significant difference was detected, some women in the case group exhibited stronger PFM contractions (*p* = 0.076), while others were able to sustain contracted PFM for a longer duration (*p* = 0.133).

Applying the ICIQ-SF, during the initial consultation and one year later, women were presented with seven questions about UI symptoms and their impact on daily life (Table 3). After the delivery, UI statistically significantly affected women in the control group more often. This negatively influenced the daily lives of women who belonged to the control group more often than those of women from the case group. One year later, the results regarding the complaints related to UI did not differ.

Assessment of FI before pregnancy, during the first six months postpartum, and two years after labor were conducted using the Wexner Fecal Incontinence Scale. No statistically significant difference between groups was observed. Two years after delivery, gas incontinence was more common in the case group (*p* = 0.019). One year after the initial consultation, gas incontinence was also more significantly common in the case group (*p* = 0.037). However, one year later, the total number of gas incontinence cases were reduced from five women to three in the case group.

Similar to UI, FI negatively affected the abilities of women in both groups to perform household chores, engage in social interactions, and maintain psychological well-being. However, no statistically significant difference was found.

## 4. Discussion

Perineal tears occurring during childbirth, especially those of 3rd or 4th degree, are correlated with an increased risk for pelvic floor dysfunction in the future [1,4,11]. These involve various conditions, such as UI, FI, POP, altered sexual dysfunction, and chronic pelvic pain [4]. The importance of these conditions emphasizes the need for preventive and therapeutic approaches. PFM strengthening is recommended as a key strategy in many international guidelines dedicated to the problem of incontinence or pelvic floor dysfunction [1,5,6].

The main risk factors for perineal tears in our study were fetal macrosomia, fetal occiput posterior position, and epidural analgesia. Similar results were noted in other studies [4,11,12,13]. V. L. Longo et al. investigated a broad range of antenatal and intrapartum risk factors for 3rd- and 4th-degree perineal tears, including the risk factors mentioned above [14]. In addition to the mentioned risk factors, the number of obstetric examinations during the delivery (more than five per delivery), patient position at delivery (especially lying position), and instrumental delivery were identified. Instrumental delivery is a well-known risk factor for OASIS, especially in cases where forceps are used [15].

There were no statistically significant differences found when comparing participants’ anthropometric data (weight, height, and BMI). Similar data were reported in a study conducted by K. Gallagher et al. Neither a woman’s BMI nor excessive weight gain during pregnancy increased the risk for perineal trauma [16]. However, a recent study conducted by A. Fruscalzo et al. drew conclusions indicating that nulliparity and feto-maternal BMI stand out as the two most reliable predictors of 3rd- and 4th-degree perineal lacerations [17].

The reported prevalence of UI in the postpartum period ranged from 3 to 40% [18]. The use of ICIQ-SF [5] in our study allowed us to evaluate symptoms associated with UI and their effects on the daily lives of participants. In our study, over half of the women were affected by UI after delivery. These results are in accordance with existing literature that emphasize the connection between 3rd- or 4th-degree perineal tears and an elevated risk of UI [18]. On the contrary to the expected outcomes, the control group exhibited an increased prevalence of UI during the study period. Similar results were presented by L. Lawrence in 2016, where perineal trauma was not associated with urinary or fecal incontinence, decreased sexual activity, perineal pain, or pelvic organ prolapse [19]. In our study, several women in both groups reported experiencing emotional well-being implications and feelings of disappointment associated with UI. This qualitative aspect added depth to the understanding of the subjective experience of UI, emphasizing the importance of addressing not only the physical symptoms but also the psychosocial impact on women’s lives [20].

OASIS at the time of vaginal delivery increases the risk of FI [21]. Our study found that there was a notable trend in the incidence of gas incontinence in case groups two years after delivery and one year after the initial consultation. The systematic review performed by N. A. Okeahialam et al. concluded that an increasing degree of sphincter injury is associated with poorer anal incontinence outcomes [22]. A scientist from Sweden concluded that not only sphincter tear but also vaginal delivery itself is a risk factor for gas or fecal incontinence after birth [23]. Various studies found that FI is also a psychosocially debilitating disorder that negatively impacts quality of life [24,25].

The primary objective of our study was to educate women about PFM strengthening exercises. Education is significantly important, considering the deficiency in awareness and information regarding these exercises in Lithuania. The knowledge gap underscores the necessity for comprehensive postpartum care, which should include education on PFM exercises [26]. In literature, the main causes for not performing PFM exercises are lack of time, lack of motivation, and not seeing immediate results. Such education aims to empower women in effectively managing and preventing pelvic floor dysfunction [26,27]. The conclusion drawn by P. B. Tchounwou et al. asserts that furnishing information and foundational concepts about the pelvic floor and offering verbal instructions to guide women in performing correct PFM contractions enhance their PFM contractile capability [28]. It is found that optimal outcomes in PFM strengthening are realized when woman is regularly supervised by a specialist [8].

Moreover, there are other alternative methods which could be used to treat UI. One of them is neuromuscular electrical stimulation (NMES), particularly for stress UI. Research studies have demonstrated the efficacy of NMES in enhancing PFM strength, endurance, and coordination, ultimately leading to improvements in UI and quality of life for individuals affected by UI. NMES offers a non-invasive and convenient treatment option, making it suitable for a wide range of patients, including those who may not be candidates for surgical interventions or pharmacotherapy [29]. Other authors state that PFM exercises combined with NMES have a good therapeutic effect on postpartum pelvic floor dysfunction, which can markedly improve PFM strength and vaginal pressure [30].

One of the primary obstacles encountered during this study was the recruitment and retention of participants, particularly those who had experienced 3rd- and 4th-degree perineal tears. Given the low incidence rate of such tears (0.4% in 2021), finding a sufficient number of eligible participants was challenging. Additionally, the sensitive nature of the topic might have deterred some women from participating or continuing with the study. The disparity in the number of consultations between the case group (three consultations) and the control group (two consultations) could have also contributed to variations in adherence and data consistency. Furthermore, ensuring that all participants received and followed the PFM strengthening instructions correctly posed a significant obstacle, as improper execution could skew the results.

The study faced several limitations that could affect the generalizability and robustness of its findings. First of all, the relatively small sample size, particularly in the case group, limits the power of the study and the ability to detect statistically significant differences. To address the relatively small sample size, the study was designed with proactive recruitment strategies, including multiple follow-up attempts via phone calls to maximize participant retention and adherence. Another limitation was that the transvaginal ultrasound probe was used to monitor the healing process instead of the transperineal sonography probe. The observational nature of the study also means that causality cannot be firmly established between PFM strengthening and improved pelvic floor function. Additionally, to mitigate the potential bias introduced by self-reported data, validated questionnaires (ICIQ-SF and Wexner Fecal Incontinence Scale) were used, and participants were encouraged to provide honest and thorough responses. Lastly, the follow-up period, while substantial, may still be insufficient to capture long-term outcomes and variations in pelvic floor function post-intervention. These steps collectively aimed to enhance the study’s validity and reliability, despite the inherent limitations.

## 5. Conclusions

This study found that PFM strengthening exercises significantly improved pelvic floor function in women who experienced OASIS two years after delivery. Participants exhibited stronger PFM contractions and an increased ability to maintain these contractions. Women reported a better understanding of PFM exercises and proper techniques.

## Figures and Tables

**Table 1:** Sociodemographic data.

Criteria		Case Group	Control Group	*p*-Value
		*n* = 10	*n* = 7	
Patient age		34 (30–44; 5.2) ^1^	31 (26–34; 2.8) ^1^	0.024 ^2^
Place of residence	Urban, *n* (%)	5 (50)	5 (71.4)	0.179
	Rural, *n* (%)	5 (50)	2 (28.6)	0.214
Education	Primary, *n* (%)	1 (10)	0 (0)	0.854
	Secondary, *n* (%)	2 (20)	1 (14.3)	0.623
	Higher, *n* (%)	7 (70)	6 (85.7)	0.222
Marital status	Married/partnership, *n* (%)	9 (90)	6 (85.7)	0.377
	Single, *n* (%)	1 (10)	1 (14.3)	0.396

^1^ Median (min–max; SD). SD, standard deviation. ^2^ *p* < 0.05, statistically significant difference.

**Table 2:** Risk factors for 3rd- or 4th-grade perineal tears.

Criteria	Case Group	Control Group	*p*-Value
	*n* = 10	*n* = 7	
Fetal macrosomia, *n* (%)	6 (60.0)	1 (14.3)	0.012 ^1^
Fetal occiput posterior position, *n* (%)	5 (50.0)	0 (0)	0.001 ^1^
Instrumental delivery, *n* (%)	1 (10.0)	1 (14.3)	0.396
Primipara, *n* (%)	5 (50.0)	4 (57.1)	0.581
Epidural analgesia, *n* (%)	9 (90.0)	2 (28.6)	0.003 ^1^

^1^ *p* < 0.05, statistically significant difference.

**Table 3:** Questions related to UI during the initial consultation.

Criteria		Study Group	Control Group	*p*-Value
		*n* = 10	*n* = 7	
UI before pregnancy, *n* (%)		1 (10.0)	1 (14.3)	0.396
UI after delivery, *n* (%)		4 (40)	5 (71.4)	0.046 ^1^
UI duration after delivery	1 month, *n* (%)	0 (0)	1 (14.3)	0.140
	2 months, *n* (%)	1 (10.0)	0 (0)	0.149
	3 months, *n* (%)	1 (10.0)	1 (14.3)	0.396
	6 months, *n* (%)	1 (10.0)	0 (0)	0.149
	Currently persisting problem, *n* (%)	1 (10.0)	3 (42.9)	0.038 ^1^
UI currently	Once a week or less, *n* (%)	1 (10.0)	4 (57.1)	0.012 ^1^
	2–3 times a week, *n* (%)	2 (20.0)	0 (0)	0.057
	Once or several times a day, *n* (%)	1 (10.0)	0 (0)	0.146
Impact on daily life, scores (SD)		2.4 (2.9)	3.6 (2.9)	0.020 ^1^

^1^ *p* < 0.05, statistically significant difference. SD, standard deviation.

## Data Availability

Anonymized data used for the study are stored on a separate biomedical study data storage computer that was used to conduct the biomedical study. Data will be stored for 15 years after the study and later destroyed.
